# Infant formula with cow’s milk fat and prebiotics affects intestinal flora, but not the incidence of infections during infancy in a double-blind randomized controlled trial

**DOI:** 10.1186/s40348-020-00098-1

**Published:** 2020-07-02

**Authors:** Antonia Nomayo, Andreas Schwiertz, Rainer Rossi, Katharina Timme, Janine Foster, Richard Zelenka, Josef Tvrdik, Frank Jochum

**Affiliations:** 1 Department of Pediatrics, Evangelisches Waldkrankenhaus Spandau, Stadtrandstr. 555, 13589 Berlin, Germany; 2 Institute of Microecology, Herborn, Germany; 3 Department of Pediatrics, Vivantes Klinikum Neukölln, Berlin, Germany; 4 DMK Baby GmbH, Bremen, Germany; 5 Department of Computer Sciences, University of Ostrava, Ostrava, Czech Republic

**Keywords:** Beta-palmitic acid, Microbiota, Bifidobacteria, Galactooligosaccharides, Immunity

## Abstract

**Background:**

The postnatal intestinal colonization of human milk-fed and formula-fed infants differs substantially, as does the susceptibility to infectious diseases during infancy. Specific ingredients in human milk, such as prebiotic human milk oligosaccharides and a specifically structured fat composition with high proportion of beta-palmitic acid (beta-PA) promote the growth of intestinal bifidobacteria, which are associated with favorable effects on infants’ health. The present study investigates whether addition of prebiotic galactooligosaccharides (GOS) in combination with higher amounts of beta-PA from cow’s milk fat in infant formula positively affects gut microbiota and the incidence of infections in formula-fed infants.

**Methods:**

In a double-blind controlled trial, formula-fed infants were randomly assigned to either receive an experimental formula containing a higher proportion of beta-PA (20–25%) from natural cow’s milk fat, and a prebiotic supplement (0.5 g GOS/100 ml), or a standard infant formula with low beta-PA (< 10%), without prebiotics. A breast-fed reference group was also enrolled. After 12 weeks, fecal samples were collected to determine the proportion of fecal bifidobacteria. The number of infections during the first year of life was recorded.

**Results:**

After 12 weeks, the proportion of fecal bifidobacteria was significantly higher in infants receiving formula with high beta-PA and GOS compared to control, and was similar to the breast-fed group (medians 8.8%, 2.5%, and 5.0% respectively; *p* < 0.001). The incidence of gastrointestinal or other infections during the first year of life did not differ between groups.

**Conclusions:**

The combination of higher amounts of beta-PA plus GOS increased significantly the proportion of fecal bifidobacteria in formula-fed infants, but did not affect the incidence of infections.

**Trial registration:**

The study protocol was registered with Clinical Trials (Protocol Registration and Results System Trial ID: NCT01603719) on 05/15/2012 (retrospectively registered).

## Background

Human breast milk with its optimally balanced nutrient composition and its various bioactive components is the ideal nutrition for infants during the first months of life. For infants who are not able to receive human milk (HM), constant improvement of human milk surrogates is desirable. Infant nutrition has a major impact on health and human breast milk offers many health benefits: among other advantages, breast-fed infants have a lower incidence of infectious diseases than their formula-fed counterparts [1–4]. In addition to various other ingredients, human milk oligosaccharides (HMOs), originally identified as the “bifidus factor” in human breast milk, are believed to contribute to this benefit by supporting the growth of health-promoting bifidobacteria [1, 2, 5]. This presumably has positive effects on intestinal maturation and immunity development [6, 7]. A fecal flora dominated by bifidobacteria is more common in breast-fed than in formula-fed children, while the latter are more likely to have a more diverse “adult” type intestinal microbiota. These differences in bacterial colonization have been demonstrated using both molecular and culture-based detection techniques [1, 8–10].

HMOs are structurally complex sugar molecules from the group of oligosaccharides that occur almost exclusively in human milk. They have no nutritional, but great functional value for the infant. Approximately 200 structurally different HMOs have been detected to date in human breast milk, with strong inter-individual variability, and in concentrations between about 5 and 15 g/l [5, 11, 12]*.* HMOs can be metabolized particularly well by bifidobacteria [5] and therefore exert prebiotic, bifidogenic effects*.* Most conventional cow’s milk-based formulas do not contain HMOs, since the addition of synthetically produced HMOs to infant food has only recently become subject to clinical testing [13].

In bottle-fed infants, the growth of bifidobacteria can be stimulated by addition of prebiotic supplements such as galactooligosaccharides (GOS) to infant formula [14–22].

Recent investigations also address the influence of triglyceride structure in infant milk on the intestinal microbiota [23, 24]. With the aim of developing infant formula with a fat mixture that better resembles human milk fat, there has been increasing interest in the fat composition and structure in infant milk fat in recent years [25–27]. The growing infant takes in approximately half of its energy needs from the lipids contained in human breast milk or HM substitutes, with almost all milk fat being provided in form of triacylglycerides (TAGs) [26, 28]. TAGs are composed of 3 variable fatty acids esterified to a glycerol backbone. Palmitic acid (PA) is the main fatty acid, both in TAGs of human breast milk as well as in synthetic infant milk [26, 28]. However, stereospecific distribution of PA within the triglyceride molecule differs substantially between HM fat and fat blends used in conventional infant formula, depending on the fat sources used [25, 26, 29]. In HM, high proportions (up to over 70%) of PA are esterified to the beta-(center-)position, whereas in most infant formula with fat sources mainly derived from vegetable oils, proportions of beta-palmitate are very low [25]. Stereospecific positioning within the TAG molecule has important impact not only on digestion and absorption of the long-chain saturated PA from infant milk [29, 30], but also on the absorption of dietary calcium. Free palmitic acid that is not absorbed is prone to form insoluble calcium soaps that are excreted into feces [27]. This leads to lower availability of calcium and fat and probably causes adverse effects to the infant, such as increased stool hardness, constipation, infantile colic, and changes to the intestinal microenvironment [27, 31]. Increasing the proportions of beta-palmitate by using artificially structured lipids in infant formula has shown to have potentially beneficial effects in clinical studies. It was associated with improved fatty acid and calcium absorption, decreased formation of calcium-fatty acid soaps in the feces, and softer stools in formula-fed infants (for an overview see [27]). In addition to those biochemical effects, increased beta-PA concentration in infant formula also resulted in an increase of bifidobacteria growth in the gut of formula-fed infants [23, 24]. However, in a recent position paper by the ESPGHAN (European Society for Paediatric Gastroenterology, Hepatology, and Nutrition) Committee on Nutrition, the existing evidence in regard to the clinical effects of dietary high beta-PA in infant formula was evaluated. In summary, the inclusion of high beta-PA in infant formula was considered as not essential, mostly due to the lack of high-quality evidence on relevant clinical benefits, and further research on health effects of beta-PA-based infant formulas was demanded [32]. In this clinical study, an experimental cow’s milk-based infant formula was designed that contained both the addition of GOS and a modified fat body with an increased concentration of natural beta-PA from a mixture of butterfat with vegetable and fish oils. Cow’s milk fat naturally contains higher concentrations of beta-palmitate (approx. 40% of the fat content in TAGs) than fat mixtures from vegetable oils [29], without the need for artificial inter-esterification.

Our basic idea was to develop and scientifically test a breast milk surrogate with a natural source of beta-PA from cow’s milk fat combined with an established prebiotic additive in order to support the development of a beneficial micromilieu in the intestine of formula-fed infants, but without having to resort to more expensive fat sources with highly concentrated beta-PA. Accordingly, it was our goal to observe a possible synergistic effect of the combination of two functional agents.

We hypothesized that the experimental formula would (i) induce an increased proportion of bifidobacteria in feces and (ii) reduce the incidence of (gastrointestinal) infections during infancy.

As safety parameter formula intake and body growth were recorded.

Additional observations included evaluation of gastrointestinal tolerance, as well as biochemical stool and blood analyses, which are not subject of the present report, but are evaluated and will be reported elsewhere.

## Methods

### Study design and proceedings

In a prospective randomized double-blind controlled trial, two groups of formula-fed newborns were fed with one of two study formula. Additionally, a non-randomized, breast-fed (BF) reference group was included.

Participants were recruited at three maternity units in Berlin, Germany, between August 2011 and August 2013. Eligible infants (birth weight 10th to 90th percentile according to Voigt et al. [33], term birth ≥ 37 gestational weeks, apparently good health, parents’ written consent) were enrolled within their first 10 days of life (DOL). For the formula groups, only mothers who had independently decided against breastfeeding were addressed. Inclusion criterion for enrollment in the BF group was the intention to breastfeed for at least 3 months. Exclusion criteria were any disorders which may influence growth or type of feeding, systemic antibiotic treatment prior to enrollment, and a family history of atopic disease. Intake of additional pre- or probiotic supplements was not allowed during the intervention period.

The formula groups were randomized using a pre-prepared, computer-generated randomization list (random permuted block design, blocks of 4). The two study formula compositions were coded using capital letters (A, B, C, or D) with each formula type being labeled by either 2 of the 4 letter codes. Formula production, packaging, and coding were performed by the manufacturer. Investigators, study personnel, and parents were blinded to the encoding until completion of data analysis.

The experimental formula was supplemented with 0.5 g/100 ml GOS and contained a unique fat blend combining cow’s milk fat, vegetable oils, and fish oil to achieve a 20–25% proportion of beta-PA. No artificially structured lipid sources were used in the experimental formula. The control formula did not contain any prebiotic supplement; the fat blend predominately contained vegetable oils with a considerably lower proportion of beta-PA (< 10%). Apart from those alterations, the compositions of the study formula products were comparable (for main components see Table 1).

**Table 1:** Study formula composition

		HbPA+ formula	Control formula
Energy	kcal/100 ml	64	65
Protein	g/100 ml	1.4	1.4
	g/100 kcal	2.1	2.1
Carbohydrate	g/100 ml	7.3	7.8
GOS	g/100 ml	0.5	0
Fat	g/100 ml	3.1	3.1
PA (16:0)	mg/100 ml	769 (24.8%)	693 (22%)
Beta-PA		20–25%	< 10%

*PA* palmitic acid; *GOS* galactooligosaccharides; *HbPA+ formula* formula supplemented with galactooligosaccharides and fat blend with cow’s milk fat, vegetable, and fish oil (modified to contain 20–25% of beta-palmitic acid); *control formula* formula not containing GOS, fat blend containing vegetable, and fish oil without cow’s milk fat

Study formula was fed for at least 12 weeks, but maximally to the onset of weaning, usually at the age of 5–6 months.

Infants in the BF group were predominantly breast-fed with less than 20% supplementary formula consumption.

Data acquisition comprised socioeconomic and medical data, and fecal samples at enrollment, followed by two study visits with physical examinations, anthropometric measurements, parental interviews, and collection of fecal samples at 6 and 12 weeks of life. Parents filled in 3-day protocols on drinking volumes and food tolerance prior to each study visit. Structured telephone questionnaires were performed between visits. After the 12-week intervention, parents kept a parental diary for further documentation of the medical course with focus on infections during the first year of life.

Primary outcome measures were the number of gastrointestinal infections during the first year of life, and the proportion of bifidobacteria in feces after 12 weeks. Acute gastrointestinal infections were defined as three or more watery stools per day plus change of smell or color and/or additional signs of illness (fever, vomiting, and irritability). Respiratory tract infections were defined as the onset of rhinitis, coughing, or wheezing with or without fever. Infectious episodes (gastrointestinal or respiratory) were documented by the parents based on the aforementioned criteria, or based on a physician’s diagnosis.

To determine the proportion of bifidobacteria, fecal samples were filled from the child’s diaper into collection tubes and brought to the study visits either fresh or frozen. The samples were then stored at − 80 °C until analysis. Fecal microbiota was analyzed by quantitative real-time PCR using selective primers to recognize the genus *Bifidobacterium* and total bacteria as previously described [34, 35]. Real-time PCRs were performed in triplicate, average values were used for enumeration. Proportion of bifidobacteria was defined as the quotient between bifidobacteria count and total fecal bacteria (TFB) count in a fecal sample of 1 g of dry weight.

Weight, recumbent length, and head circumference were measured by trained personnel during each visit using calibrated electronic infant scales, non-extractable tape measures, and a length board. Anthropometric data at birth were derived from birth records. In formula-fed infants, mean daily formula intake was calculated from the 3-day food protocols.

### Statistical analyses

Statistical computations were carried out by an investigator independent of the study group using the NCSS statistical system [36].

Sample size was calculated to detect a 20% reduction of acute gastrointestinal infections during the first year of life. With an estimated average of 0.74 (± 0.2) gastrointestinal infections within the observation period [37] and allowing for a dropout rate of 30%, the inclusion of 48 participants per formula group was intended (power 80%, due to the existence of two primary outcome parameter levels of significance was set at 0.025). This sample size was also adequate to detect differences in the proportion of fecal bifidobacteria after the 12-week intervention.

Growth data was analyzed using one-way ANOVA, followed by Tukey-Kramer multiple comparison tests. For comparison of formula intake, two-sample *t* test was used.

For the primary outcome parameters (incidence of infections, proportion of fecal bifidobacteria), group differences were analyzed using non-parametric Kruskal-Wallis one-way ANOVA on ranks. In a post hoc analysis, multiple comparison analysis tests (Bonferroni) were used. To put attention on participants who experienced an above average number of infections, infectious counts were dichotomized using the categories ≤ 1 or > 1 infection during the intervention period, and alternately using the categories ≤ 2 or > 2, and ≤ 3 or > 3 infections during the first year of life. Dichotomized data was analyzed using chi-squared test of independence.

To review possible confounders, baseline values of fecal bifidobacteria were tested for group differences, using Kruskal-Wallis ANOVA. Additional analyses evaluated the influence of birth mode on the proportion of bifidobacteria, using two-way ANOVA. Influence of baseline values on later bifidobacteria count was tested using two-sample Wilcoxon test. Wilcoxon signed-rank test was used to evaluate a significant increase in bifidobacteria count over time within the intervention period.

## Results

A total of 94 infants were randomized to receive one of the study formula compositions (high beta-PA and GOS (hbPA+) group *n* = 47, control group *n* = 47). Two infants were excluded before receiving any study feedings due to recruitment mistakes (violation of inclusion criteria, both hbPA+ groups). To comply with the intended timeline, recruitment was stopped after the enrollment of 94 instead of 96 infants. Study participants of both formula groups showed similar distribution of baseline characteristics (see Table 2, showing baseline characteristics and safety parameters). Possible confounders on the primary outcomes, like gestational age, perinatal antibiotic treatment, and birth mode were evenly distributed with no significant differences between groups, except that parents in the breast milk group significantly more often had higher school education (chi-squared statistics 8.5314, *p* value 0.014), and BF group tended to have higher proportion of children delivered by cesarean section (CS) than both formula groups (not statistically significant). Thirty-four breast-fed infants were enrolled in the non-randomized BF group. Attrition was considerably higher than estimated; 39% of the study participants in the formula groups (hbPA+ = 36%, control = 43%) and 47% in the BF group discontinued the study before 12 weeks. Fifty-seven infants (hbPA+ *n* = 30, control *n* = 27) in the formula group and 18 infants in the BF group completed the intervention period with evaluable data on primary outcome parameters. In two cases (hbPA+ group), fecal samples could not be obtained. Formula-fed infants dropped out mainly for changing to another formula (*n* = 25), or for not attending visits (*n* = 7). Main reason for dropout in the BF group was discontinuation of breastfeeding (*n* = 9) and not attending visits (*n* = 6).

**Table 2:** Baseline characteristics and safety parameters according to feeding group^§^

	HbPA+ group	Control group	BF group
**Baseline characteristics**	*n* = 31	*n* = 27	*n* = 18
Gender m/f	15/16	16/11	10/8
Mean birth weight ± SD (g)	3287 ± 377	3354 ± 376	3520 ± 444
Mean gestational age ± SD (weeks)	39.2 ± 1,2	39.6 ± 1,2	39.7 ± 1.3
Cesarean delivery, *n* (%)	6 (19)	5 (19)	6 (33)
Antibiotic treatment prior to birth, *n* (%)	1 (3)	2 (7)	2 (11)
Parent with higher education entrance qualific. (Abitur), *n* (%)	9 (29)	4 (15)	10 (56)*
**Anthropometrics**	*n* = 31	*n* = 27	*n* = 18
Mean weight gain ± SD after 12 weeks (g/d)	31.2 ± 9.5	32.6 ± 10.1	27.9 ± 8.4
Mean length gain ± SD after 12 weeks (cm/d)	0.11 ± 0.02	0.11 ± 0.03	0.09 ± 0.03**
Mean head growth ± SD after 12 weeks (cm/d)	0.06 ± 0.01	0.06 ± 0.01	0.06 ± 0.01
**Formula consumption**	*n* = 30	*n* = 27	
Mean formula intake at 6 weeks (ml/kg bw)	155 ± 30	164 ± 30	n.a.
Mean formula intake at 12 weeks (ml/kg bw)	134 ± 26	134 ± 21	n.a.

^§^Participants who have completed the intervention period

*m* male, *f* female, *SD* standard deviation, *bw* body weight, *HbPA+ group* participants receiving formula with high beta-PA and GOS supplement, *control group* participants receiving standard infant formula, *BF group* participants predominantly breast-fed, *n.a.* not applicable

*There was significant difference in proportion of parents with higher education level between feeding groups (*p* value .014); **difference in mean length gain after 12-week intervention was significantly lower in breast-fed infants compared to infants in control formula group

**Fig. 1:**
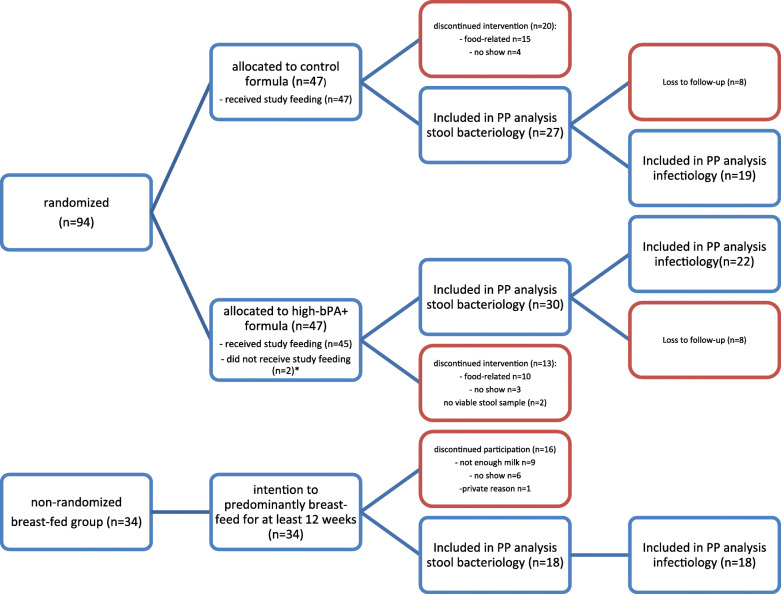
Flow diagram of study participants. HbPA+ formula = formula supplemented with galactooligosaccharides and fat blend with cow’s milk fat, vegetable, and fish oil (modified to contain 20–25% of beta-palmitic acid); control formula = formula not containing GOS, fat blend containing vegetable, and fish oil without cow’s milk fat. *2 participants in the hbPA+ group were retrospectively excluded due to recruitment error (did not meet inclusion criteria). **analysis of secondary endpoints “infection rates during 12-week intervention”

Forty-one infants in the formula groups (verum 47%, control 40%) and 18 infants in the BF group (53%) completed the follow-up period (first year of life) (for flow diagram of participants, see Fig. 1).

### Microbial analysis of feces

**Fig. 2:**
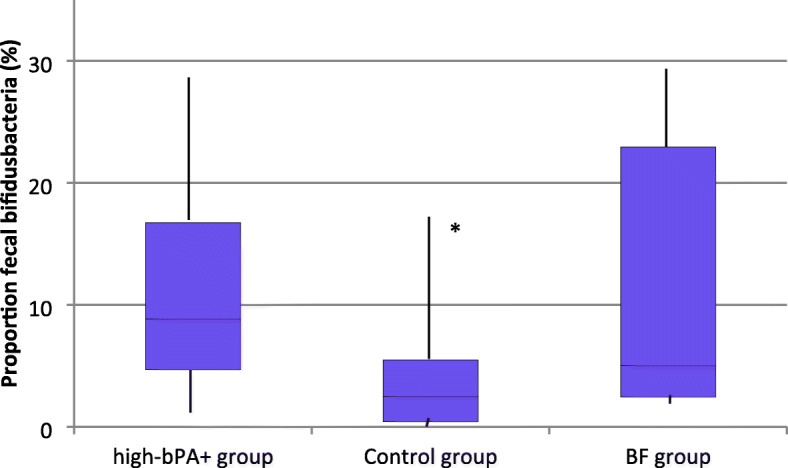
Stool microbiota. Boxplots of median percentages of fecal bifidobacteria on total stool bacteria among feeding groups. Stool microbiota was determined by real-time PCR using selective primers to recognize the genus Bifidobacterium and total bacteria. High-bPA+ group = participants receiving formula with high beta-PA and GOS supplement, control group = participants receiving standard infant formula, BF group = breast-fed reference group; *percentage of bifidobacteria in the control formula group was significantly lower than in the hbPA+ formula and breast milk groups (*p* = 0.0005), respectively; difference between breast milk and verum formula group was not significant

In respect to the proportion of bifidobacteria, a significant difference between feeding groups was observed following intervention (medians for hbPA+, control, and BF group were 8.8%, 2.5%, and 5%, respectively; *p* = 0.0005). Proportion of bifidobacteria was significantly higher in the hbPA+ group compared to control. However, it did not differ between hbPA+ and BF group (see Fig. 2). Total fecal bifidobacteria count (BBC) was also higher in the hbPA+ and BF groups in comparison to control (medians (± MAD) 4.4 (± 5.4) × 10^8^, 7.3 (± 10.8) × 10^8^, and 0.7 (± 2.1) × 10^8^, respectively; *p* = 0.00001; see Table 3).

**Table 3:** Fecal bifidobacteria at enrollment and after intervention

	BBC baseline	Bif percent baseline	BBC 12 weeks	Bif percent 12 weeks
**Fecal bifidobacteria mean (± SD)**				
HbPA+ group, *n* = 30	1.8 (± 3.5) × 10^8^	2.9 (± 4.9) %	6.7 (± 7.8) × 10^8^*	11.3 (± 8.8) %*
Control group, *n* = 27	2.4 (± 6.5) × 10^8^	4.4 (± 7.6) %	2.1 (± 3.6) × 10^8^	4.3 (± 4.9) %
BF group, *n* = 18	4.2 (± 8.4) × 10^8^	4.5 (± 7.7) %	12.9 (± 18.8) × 10^8^*	12.1 (± 11.0) %*
**Fecal bifidobacteria median (± MAD)**				
HbPA+ group, *n* = 30	0.3 (± 2.2) × 10^8^	1.0 (± 3.1) %	4.4 (± 5.4) × 10^8^*	8.8 (± 7.1) %*
Control group, *n* = 27	0.3 (± 3.4) × 10^8^	0.8 (± 5.2) %	0.7 (± 2.1) × 10^8^	2.5 (± 3.8) %
BF group, *n* = 18	0.5 (± 5.6) × 10^8^	1.5 (± 5.0) %	7.3 (± 10.8) × 10^8^*	5.0 (± 10.2) %*

Table shows development of fecal bifidobacteria from enrollment until the end of the 12-week intervention.

*BBC* absolute bifidobacteria count, *Bif percent* proportion of bifidobacteria to total fecal bacteria (percentage), *SD* standard deviation, *MAD* mean absolute deviation, *hbPA+ group* group receiving formula with high beta-PA concentration and GOS supplement, *control group* standard formula group, *BF group* breast-fed group

*Median number at baseline (total bifidobacteria count and/or percentage of bifidobacteria) differs significantly from numbers after intervention according to Wilcoxon signed-rank test (*p* < 0.001)

Distribution of fecal bifidobacteria at enrollment was comparable between the formula groups. However, following intervention infants in the hbPA+ group showed a significant increase both in BBC, as well as in the proportion of bifidobacteria to TFB (*p* < 0.0001 and *p* = 0.0007, respectively). In contrast, infants in the control group did not show those changes (see Table 3).

As a possible confounding factor, antibiotic use in the study participants was recorded. We found that antibiotic treatment was carried out very rarely and therefore did not appear to play a role as a confounding factor. As few as 2 infants (both hbPA+ group) underwent antibiotic treatment within the intervention period, overall only 8 infants in the entire study population received antibiotic treatment during their first year of life (4 in the hbPA+ group, 3 in the control group, 1 in the BF group).

To examine the influence of birth mode, fecal microbiota of infants after CS were compared to infants after vaginal delivery. At enrollment, but in the formula-fed groups only, infants delivered by CS had significantly lower proportion of fecal bifidobacteria compared to infants after vaginal delivery (*p* = 0.0019). This difference had vanished after the intervention. Within the HM-fed reference group, no influence of birth mode was observed.

### Infectiology

No significant difference between feeding groups was found in regard to the primary endpoint number of gastrointestinal infections during the first year of life. Taken together, the number of gastrointestinal infections was surprisingly low in the study population. The vast majority of the children did not experience one single episode of diarrhea during their first months of life, and after all, over 60% of the participants had not experienced gastrointestinal infection by the end of their first year of life. Likewise, no significant difference in additional infection parameters was found (number of respiratory infections and total number of infections). As overall incidence of infections was low within all study groups (see Table 4, showing median number of infections), we examined whether the intervention influenced the number of children presenting with more infectious episodes than usual. Infectious counts were dichotomized using the categories of exceeding or not exceeding the median number of infections during the intervention period, or during the 1-year follow-up, respectively. At the end of the intervention, fewer children in the BF group had more than 2 episodes of respiratory infections compared to the control group (*p* = 0.04). No further significant differences between groups were observed.

**Table 4:** Infectiology—table of medians

**Number of events: median (interquartile range Q1–Q3)**	**hbPA+ group, *n* = 31**	**Control group, *n* = 27**	**BF group, *n* = 18**	*p* value*
GI at 12 weeks	0 (0–0)	0 (0–0)	0 (0–0)	0.46
RTI at 12 weeks	0 (0–1)	1 (0–2)	0 (0–0.75)	0.18
TI at 12 weeks	0 (0–2)	1 (0–2.5)	0 (0–1)	0.14
	**hbPA+ group, *n* = 22**	**Control group, *n* = 19**	**BF group, *n* = 18**	
GI at 1 year	0 (0–1)	0 (0–1)	0 (0–0)	0.75
RTI at 1 year	3 (1.25–4)	2 (1–5)	2 (1–3)	0.65
TI at 1 year	3 (1.25–4)	2 (1–5.5)	2 (1–3.75)	0.63

*No statistically significant difference in the number of infections (medians) among feeding groups was found (Kruskal-Wallis ANOVA with correction for ties)

*GI* gastrointestinal infections, *RTI* respiratory tract infection, *TI* total (respiratory and gastrointestinal) infection, *hbPA+ group* group receiving formula with high beta-PA concentration and GOS supplement, *control group* standard formula group, *BF group* breast-fed group

### Safety parameters

No significant difference was found between the feeding groups regarding weight gain and head growth at the end of the intervention period. However, a group difference was found (*p* < 0.05) in length growth, with children of the BF group gaining less length in comparison to control (mean length gain 0.09 cm/day vs. 0.11 cm/day). Mean formula consumption at 6 and at 12 weeks was comparable in both formula groups with no significant group differences at any time (see Table 2). No severe adverse events were reported by the parents.

## Discussion

Bacterial colonization after birth with so-called pioneer bacteria like bifidobacteria plays an important role in the development of the newborn intestine and the maturation of the immune system. The interaction of host epithelial and immune cells with the commensal intestinal microbiota during the first stages of immunity development helps to establish a natural balance of pathogen defense and immune tolerance in the host (for an overview see e.g., [6]). During the first days, following birth microbial composition is strongly influenced by various perinatal factors, such as maternal bacterial flora, birth mode, perinatal exposure to antibiotics, and the type of diet [6, 38]. The intestinal microbiota of formula-fed children differs significantly from breast-fed infants, with the latter frequently developing a stool flora rich in bifidobacteria [10]. In a large number of previous trials, various prebiotic supplements in formula foods for bottle-fed infants have been used already to prove the bifidogenicity of GOS alone, FOS, GOS/FOS mixtures, and others [14–22, 39]. Dietary stimulation of bifidobacteria growth bears the hope to exert beneficial clinical effects to formula-fed infants similar to the health benefits obtained from human milk feeding, e.g., in terms of infection protection and prevention of atopic diseases [7, 40, 41]. Also, manufacturers increasingly tend to add prebiotic supplements to infant formula products, despite ambiguities regarding the actual clinical effects of these interventions have repeatedly been pointed out by specialist bodies [42, 43] and in systematic reviews (e.g., [41, 44]). The supplementation of infant formula with prebiotics, however, has been identified to be an important field of further research, taking into account the limited available evidence on optimal doses, intake durations, combined effects with additional functional ingredients, as well as the clinical relevance of previous findings [41, 42].

In the present trial, we could show that a new infant formula containing a fat blend enriched with natural beta-palmitate, and prebiotics (GOS) was safe and led to higher proportion of bifidobacteria in the feces of formula-fed infants after an intervention period of 3 months.

We could demonstrate that feeding the experimental infant formula led to an increase in bifidobacteria in the gut, similar to the findings in breast-fed infants.

Our results are in line with former trials that have shown that supplementation with GOS alone can effectively stimulate the growth of fecal bifidobacteria in formula-fed infants [19, 21, 22]. In a double-blind randomized controlled trial (RCT) by Fanaro et al., formula-fed infants aged 4–6 months were enrolled to receive an experimental follow-on formula with or without the addition of GOS in the dosage of 0.5 g/dl for a period of 18 weeks. Stool samples were gathered after 6 weeks and at the end of the intervention. Children fed the formula with GOS had significantly higher numbers of bifidobacteria in the stools than the control group after 6 and 18 weeks [19]. Since children were enrolled at weaning, the dietary modulation of the gut microbiota started at a late phase in the colonization process, at a time when first complimentary foods might have been introduced in many children. Various influences on bacterial composition, as well as on immune development and host defense might have already taken place. Also, influences of concomitant complementary feeding on the intestinal microflora cannot be ruled out with certainty. In another study by Ben and colleagues [21], infant starter formula supplemented with a low level of 0.24 g/dl GOS was fed over a 3-month period to term infants, beginning within the first 4 weeks of life. At the end of the intervention period, the number of intestinal bifidobacteria and lactobacilli significantly increased in the GOS-supplemented formula compared to children fed a control formula without GOS, and was similar to findings in a reference group of breast-fed infants. Additionally, growth, stool characteristics, and tolerance were recorded, showing an increase in fecal short-chain fatty acids and stool frequency, as well as a decrease of fecal pH in the GOS-supplemented infants. Clinical parameters of immunity and infection prevention had not been evaluated in those clinical trials [19, 21]. In a multicenter study by Sierra et al., the prebiotic effect of a formula containing GOS in healthy infants during the first year of life was evaluated [22]. In a double-blind RCT, infants were enrolled within the first 8 weeks of life to receive infant starter formula, and subsequently follow-on formula supplemented with 0.44 g/dl and 0.5 g/dl GOS, respectively, until the age of 12 months. After 4 months, fecal samples were collected in a subgroup of infants to perform biochemical and stool bacteria analysis. A significant increase in bifidobacteria, as well as signs of an increased fermentation activity in the feces of the GOS group was shown compared to control. Following this long-term prebiotic intervention, the number of infections and the incidence of allergic manifestations up to 12 months of age did not differ between the feeding groups, though. In the summary of these trials, no safety concerns arisen, in regard to adverse events or growth impairment, so far [19, 21, 22].

It has also been shown previously that increasing the proportion of beta-palmitate in the fat blend used in infant formula, using artificially structured lipids, has an isolated bifidogenic effect [23, 24]. Yaron et al. were able to show that a 6-week period of feeding infant formula with high concentrations of beta-PA, compared to a standard low beta-PA formula (44% vs. 14% of the PA content), led to significantly higher bifidobacteria and lactobacillus counts in the feces of term infants, similar to a reference breast-fed group [23]. Neither of the study formula contained prebiotics. In another RCT by Yao et al., the effect of infant formula with a fat blend high in beta-PA, without or in combination with varying doses of prebiotics (oligofructose), was evaluated by feeding term infants one of 4 different study formula for 8 weeks [24]. The intervention was started after 2–3 weeks of life, and stool characteristics, stool bacteriology, and food tolerance were assessed. Compared to a standard formula with low beta-PA (around 12% of PA in beta-position) and without prebiotics, in all study formula containing high beta-PA (around 36-37%), with or without addition of prebiotics, the number of bifidobacteria increased significantly compared to control formula, along with improvements in stool consistency. Both aforementioned trials used so-called “structured lipids” with highly concentrated beta-PA, obtained by artificial inter-esterification, which resulted in fat blends with increased beta-PA content of over 35% of the PA [23, 24]. To our knowledge, for the first time, in the present trial we used a formula with moderately high beta-PA from a natural fat source in combination with a prebiotic supplement of GOS, with the intention of mimicking the bifidogenic effect of HM.

Blending a fraction of natural cow’s milk fat, with a mixture of vegetable and fish oil, an approximate beta-PA proportion of 20–25% of the PA content was established without having to resort to more expensive and synthetically processed high beta-PA fat sources. With the proportion of beta-PA being considerably lower than described in the aforementioned trials, our basic idea was to combine positive effects on the intestinal micromilieu by adding a prebiotic supplement of GOS. Our intention was to induce synergistic effects of the two functional ingredients rather than to observe individual effects of a single component. As a strength of our study design, and in contrast to many preceding trials [19, 21–24], the intended stool flora modulation was implemented very early within the first 10 days postpartum so that influences of the combined intervention started at an early stage of postnatal immune development. Also, during the intervention period, study formula was fed exclusively, with no further dietary influences which might have affected the results.

Interestingly, in our trial, children in the BF group had higher proportion of fecal bifidobacteria even in baseline samples, in comparison to both formula groups, despite a higher rate of cesarean delivery (33% vs. 19% in both formula groups, difference not significant). Since fecal samples were typically collected 4–10 days after birth, this rapid establishment of a bifidobacteria-rich microbiota might be due to the pre-existence of bifidobacteria in human breast milk [45], possibly combined with the highly effective prebiotic effects of HMOs [5, 11]. Further remarkable differences in fecal microbiota were observed between formula-fed and HM-fed infants with regard to the influence of birth mode: In the formula-fed groups, neonates delivered by cesarean section (CS) had significantly lower proportions of bifidobacteria at baseline. No such difference was found in HM-fed infants, though. Birth mode has been identified as one important influence on initial bacterial colonization of the human gut, at least during the neonatal period [38, 46, 47]. Previous investigations of the gut microbiota in newborns were able to demonstrate a higher bacterial diversity and greater preference and abundance of intestinal bifidobacteria after vaginal delivery compared to cesarean deliveries, with these findings being relatively constantly proven throughout the first weeks of life [46–51]. Beyond the immediate neonatal period, and particularly after the first 3 postnatal months, however, studies provide conflicting results with regard to the influence of birth mode on intestinal bacterial composition, and the association between delivery mode and bifidobacteria colonization was not consistently shown anymore [46]. Remarkably, also in the present trial, the influence of birth mode on the microbiota was no longer observed after the 3-month intervention period. This indicates that over time, diet—along with other modulating factors—has a bigger influence on the bacterial composition than the birth mode. In the breast-fed group, however, even after very few postnatal days, possible influences of birth mode were no longer detectable, suggesting that HM feeding might be a most powerful strategy to avoid dysbiosis in the neonatal gut after CS. In conclusion of those findings, we are tempted to suggest that food-induced modulations of the gut microbiota after CS may be of special benefit to formula-fed newborns and should start immediately after birth. In addition, however, mothers should be consistently informed about the far-reaching benefits of breastfeeding over formula-feeding, especially in the context of cesarean delivery.

With the aim of connecting the bacteriologic findings to clinical outcomes, we evaluated the influence of the nutritional intervention on the incidence of gastrointestinal tract infections during infancy as a primary endpoint.

Despite a clear evidence of bifidogenicity in the experimental formula, no difference in the number of infections was found in the present trial. The incidence of gastrointestinal infections was surprisingly low in all feeding groups with no group differences. High attrition resulted in significantly less available data than expected. For that reason, our study might have been underpowered to be able to show significant effects.

However, only few studies to date found preventive effects of prebiotics in infant formula on infection outcomes [18, 39, 52]. Bruzzese et al. were able to show a reduction of gastrointestinal and respiratory tract infections by using a GOS/FOS mixture in infant formula [18]. This study was an open observational trial, which might have influenced the generated results. In a prospective, randomized controlled trial, Arslanoglu et al. found a lower incidence of respiratory tract infections and diarrhea during 6 months of intervention with a prebiotic mixture of GOS/FOS [39]. This effect seemed to last up to the age of 2 years in the participating children [52]. As the study population consisted of infants with high risk of atopy and the study formula used was a hydrolyzed hypoallergenic formula, these results might not be readily transferable to children with no a priori risk of an altered immune response. In both studies that suggested a preventive effect of the prebiotic supplementation of infant formula against infections, the addition of a prebiotic 9:1 mixture from galacto- (GOS) and fructooligosaccharides (FOS) in doses of 0.4–0.8 g/dl were used [18, 39]. Accordingly, the dosage of 0.5 g/dl GOS in our trial was roughly in that dose range of the GOS portion. However, there are indications from studies in adult patients and animal models, suggesting a possible impairment of the intestinal barrier function through the administration of FOS [53, 54], which is why we refrained from adding FOS to our study food.

Concerning the incidence of respiratory infections, there were also no differences among feeding groups in our study. Within the first year of life, as few as 2 to 3 infectious episodes were reported on average. Remarkably, while most of the study participants experienced very few respiratory infections, some infants showed outstanding series of infectious episodes. Considering this as clinically important, we analyzed if an exceeding rate of infections was more common in any of the groups. No significant differences were found between groups in the observation period of 1 year. However, during the 12 weeks of intervention, we saw a slight advantage for the breast-fed infants, with slightly less infants having serial respiratory infections. This finding is not surprising and is consistent with the common expectation that breast-fed infants are less susceptible to infectious diseases [2], especially during the time of exclusive breastfeeding.

In line with our results, also other trials failed to show reduction in gastrointestinal and respiratory tract infections in infancy, when GOS was used as the only prebiotic agent in infant formula [22, 55]. Although we could demonstrate a bifidogenic effect of the experimental formula, similar to previous studies using formula enriched with prebiotic GOS and/or high beta-palmitate [21–24], clinical relevance of these findings could not be shown.

Our study results were subject to limitations, most importantly the unsuspectedly high dropout rate. Switching of formula turned out to be a frequent reaction in the study population whenever parents discerned a potential issue on food tolerance, even though an associative relationship with the type of formula mostly remained unproven. Also, we refrained from paying for the study participation to not set false incentives against breastfeeding within the local population. Without financial compensation, to some of the parents, the efforts involved in the study proceedings seemed too demanding. Since rates and reasons for dropout were evenly distributed between both formula types, differences in tolerance seemingly played no role. Nonetheless, the informative value of the results regarding the infection outcomes was markedly weakened by the reduced number of cases for per-protocol analysis. However, it should be noted, that in comparison of all groups, not even tendency differences were discernible, neither after the intervention period nor after the follow-up period of 1 year. Relevant clinical effects of the intervention referring to infection protection therefore seem to be highly unlikely. This assumption might be of interest, when varying doses and combinations of functional agents in similar experimental setups are to be evaluated in following trials.

Both study formula compositions led to adequate growth with no severe adverse effects. Compared to control, a slightly inferior length gain in the BF group was found. As growth generally lay within normal clinical ranges in all groups, we interpreted this difference as clinically irrelevant.

## Conclusions

In conclusion, we were able to demonstrate a bifidogenic effect of an experimental infant formula containing a fat blend enriched with natural beta-palmitate, and prebiotics. However, a clinically relevant effect on infections during infancy could not be shown. Surprisingly, the superiority of HM in terms of infection protection [2] could not be confirmed in our study, though we saw a tendency of serial infections being less frequent in breast-fed infants.

Although the addition of currently tested prebiotics, and high beta-PA fat blends in infant formula seem to raise no safety concerns, clear clinical benefits have not yet been reliably demonstrated to date. This is why any new prebiotic and other functional agent, as well as their combinations in infant formula have to be evaluated in regard to their clinical efficacy and safety [32, 44].

## Data Availability

The data that support the findings of this study are available from the authors upon reasonable request and with permission of the sponsor (DMK Baby).

## Abbreviations

PA: Palmitic acid
Beta-PA: Beta-palmitic acid
GOS: Galactooligosaccharides
GOS/FOS: Galactooligosaccharides/fructooligosaccharides
HM: Human milk
HMOs: Human milk oligosaccharides
BF group: Breast-fed group
DOL: Day of life
TFB: Total fecal bacteria
hbPA+ group: High beta-palmitic acid plus galactooligosaccharide group
SF group: Standard formula group
BBC: Bifidobacteria count
CS: Cesarean section
RCT: Randomized controlled trial
